# Effect of manipulation technique using ultrasound-guided cervical nerve root block on range of motion at the shoulder joint in frozen shoulder: a retrospective study

**DOI:** 10.1186/s40634-022-00500-z

**Published:** 2022-07-06

**Authors:** Kieun Park, Masashi Matsuzaki, Mitsuji Okamoto, Akihiro Sakaki, Futoshi Ikuta

**Affiliations:** 1 PAKU Painclinic, Gitex Ascent Building 5F, 6-1-20 Miyuki-dori, Chuo-ku, Kobe, Hyogo 651-0087 Japan; 2 Sonic Japan Holdings Co., Ltd, 26-11 Maruyama-cho, Hachioji, Tokyo 192-0021 Japan; 3 Riseisha College of Medicine and Sports, 3-4-21 Jusohonmachi, Yodogawa-ku, Osaka, Osaka 532-0024 Japan; 4 School of Health Sciences, Tokyo International University, 1-13-1 Matobakita, Kawagoe, Saitama 350-1197 Japan

**Keywords:** Silent manipulation, Frozen shoulder, Ultrasound-guided cervical nerve root block, Adhesion

## Abstract

**Purpose:**

The aim of this study was to evaluate the range of motion (ROM) at the shoulder joint before and after silent manipulation.

**Methods:**

This retrospective study included all patients who underwent silent manipulation at our institution between January 2013 and December 2017. In total, 1,665 shoulders in 1,610 patients (519 men, 1,146 women; mean age 55.4 ± 8.8 years) were treated during the study period. The mean symptom duration was 6.6 ± 7.1 months. ROM at the shoulder joint was measured in flexion, abduction, and external rotation before silent manipulation and at 1 week and 1, 2, and 3 months after the procedure.

**Results:**

Mean ROM at the shoulder was 98.8° (95% confidence interval [CI] 97.9–99.8) before silent manipulation and 155.5° (154.1–156.8) after 3 months in flexion (*p* = 0.0000), 75.6° (74.5–76.8) and 152.9° (151.0–154.9), respectively, in abduction (*p* = 0.0000), and 12.7° (12.0–13.4) and 45.9° (44.4–47.4) in external rotation (*p* = 0.0000). All ROM values were significantly increased at all time points after the procedure. There were no unanticipated adverse events or serious adverse reactions.

**Conclusions:**

This study reports on the efficacy and safety of manipulation using conduction anesthesia for shoulder contractures in a large group of patients. Silent manipulation can increase ROM at the shoulder safely and effectively.

**Supplementary Information:**

The online version contains supplementary material available at 10.1186/s40634-022-00500-z.

## Background

Frozen shoulder is defined as a stiff shoulder with no known cause [1, 2] and has a reported 1-year prevalence rate of approximately 0.35% among adults aged 65 years or older [3]. Frozen shoulder can be divided into a freezing stage (stage 1), a frozen stage (stage 2), and a thawing stage (stage 3), with symptoms lasting up to 26 months [4]. During this time, the patient has limited range of motion (ROM) at the shoulder joint, which interferes with daily activities. Therefore, it is necessary to improve the ROM of the shoulder joint as soon as possible to restore the patient's ability to perform activities of daily living.

Manipulation under anesthesia is often performed when ROM does not improve with conservative treatment [2]. We have been experimenting with ultrasound-guided treatment for frozen shoulder since about 2010 [5, 6]. This treatment, subsequently named “silent manipulation” by Minagawa [7], involves manipulation of the shoulder joint under local anesthetic nerve block and has been widely used [8–10]. There have been no serious complications arising from ultrasound-guided cervical nerve root block in previous studies [8–10], and its safety has been validated [11]. However, the previous studies had small sample sizes and smaller than medium effect sizes (Cohen's d = 0.5).

Therefore, the aim of this study was to determine the extent to which silent manipulation improves shoulder joint ROM and the incidence of serious complications. Our hypothesis was that silent manipulation would significantly improve shoulder joint ROM soon after the procedure without serious complications.

## Methods

This retrospective study included all patients identified in our medical records to have undergone silent manipulation at our facility between January 2013 and December 2017. The study was approved by our institutional ethics committee.

In all cases, the diagnosis of frozen shoulder was made by the same experienced doctor. The following exclusion criteria were applied: other disease that could cause limitation of ROM at the shoulder (e.g., osteoarthritis); collagen disease affecting the joints; and poorly controlled diabetes. All patients consented to treatment with silent manipulation after receiving a detailed explanation of the procedure by the doctor.

### Silent manipulation procedure

#### Glenohumeral joint injection

**Fig. 1:**
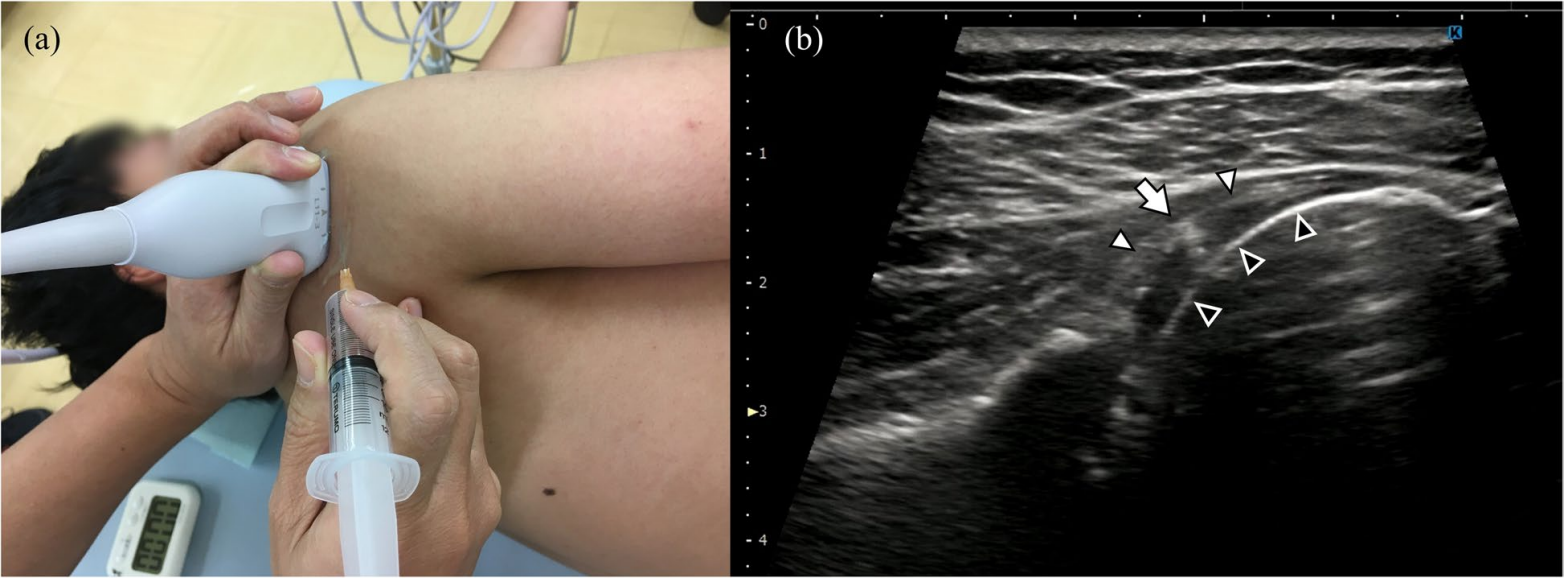
Glenohumeral joint injection. **a** With the patient in the half side-lying or lateral recumbent position, 5 mL of 1% mepivacaine and 20 mg of triamcinolone are injected into the glenohumeral joint under ultrasound guidance. **b** The white arrowhead is the capsule, the black arrowhead is the humeral head, and the white arrow is the needle tip

With the patient placed in the lateral recumbent position, the doctor identifies the glenohumeral joint using an ultrasound probe. An ultrasound-guided puncture is made and 5 mL of 1% mepivacaine and 20 mg of triamcinolone are injected (see Fig. 1, Supplemental material 1).

#### Ultrasound-guided C5 and C6 nerve root block

**Fig. 2:**
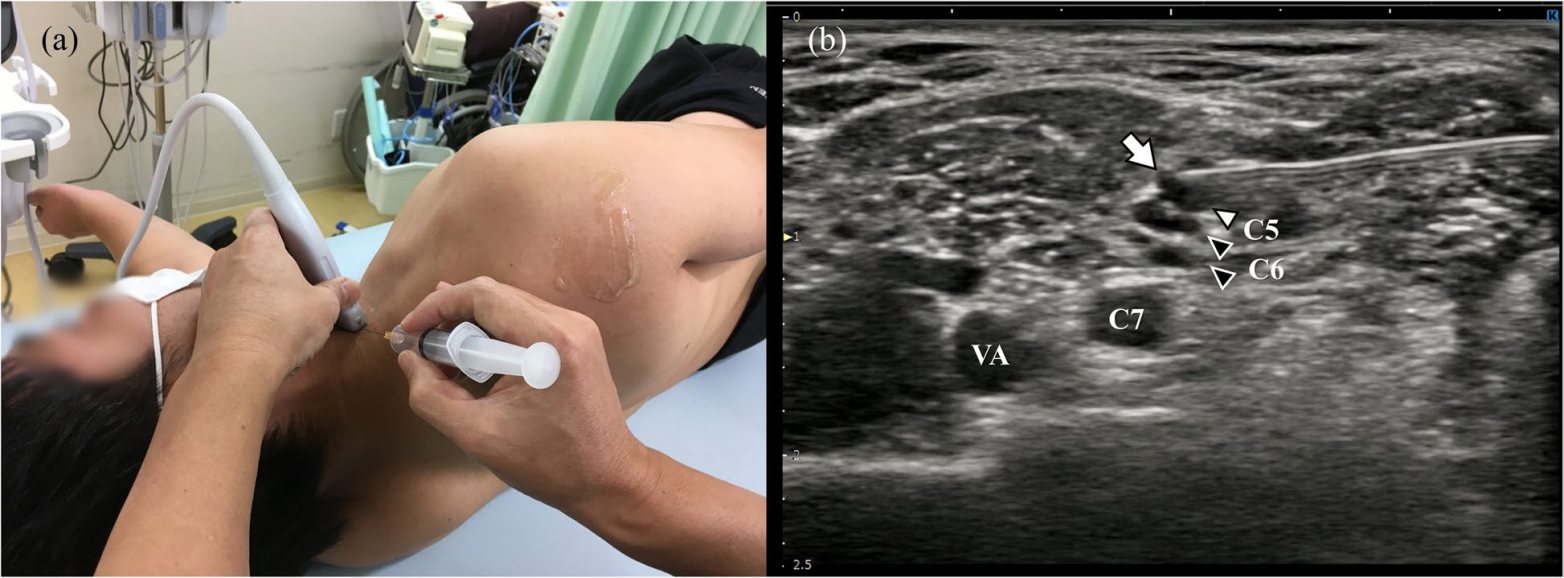
Ultrasound-guided C5 and C6 nerve root block. **a** With the patient in the half side-lying or lateral recumbent position, 12–15 mL of 1% mepivacaine is injected into the C5 and C6 nerve roots under ultrasound guidance. **b** Level where C5 and C6 form the truncus superior. The white arrowhead is C5, the black arrowheads are C6, and the white arrow is the needle tip. VA, vertebral artery

The patient is shifted to the lateral recumbent position. The ultrasound probe is placed to identify the C5 and C6 nerves [5, 7, 11], which are then blocked by injection of 12–15 mL of 1% mepivacaine under ultrasound guidance (Fig. 2, Supplemental material 2).

#### Joint manipulation

**Fig. 3:**
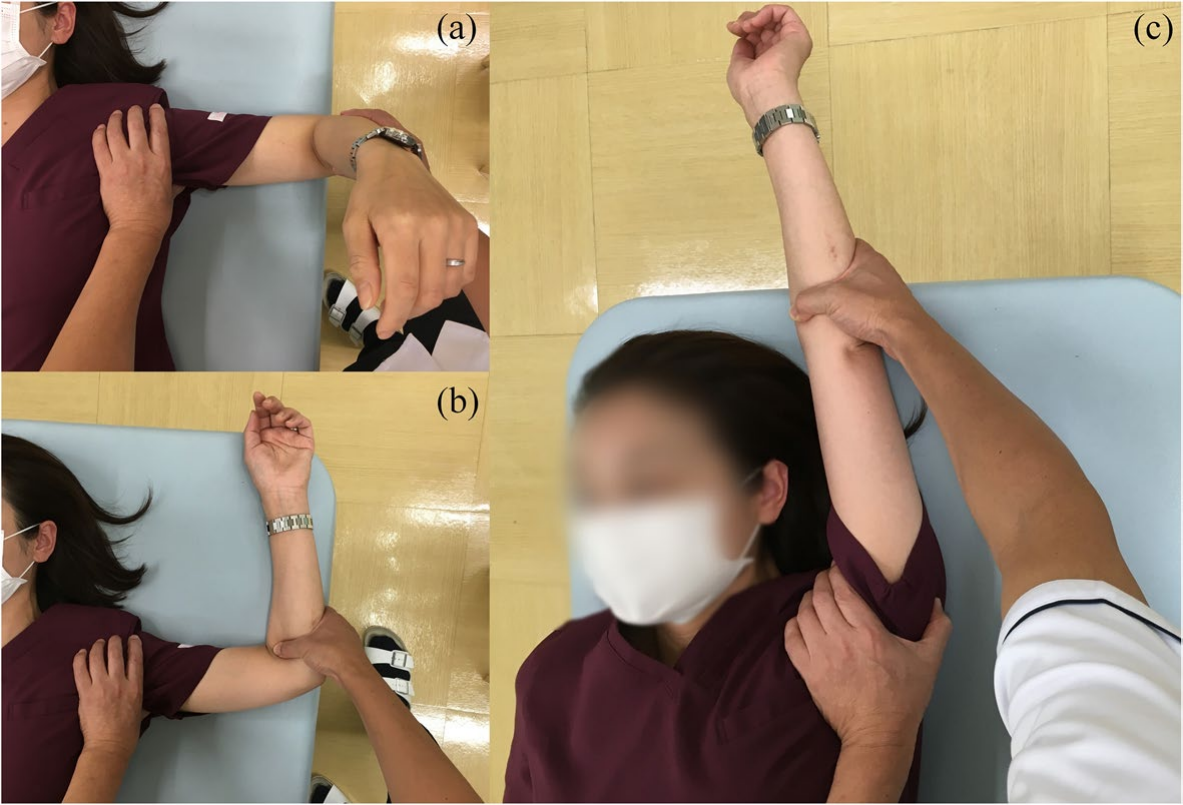
Manipulation procedure. The shoulder is abducted to 90°, then externally rotated to 90°, and maximally abducted from that position. The utmost care is taken not to fracture the humerus due to excessive external force. Care must also be taken not to move the humeral head forward when performing maximal abduction. If the joint capsule is too stiff to allow maximum abduction, it should not be forced and the operator should proceed to the next step

**Fig. 4:**
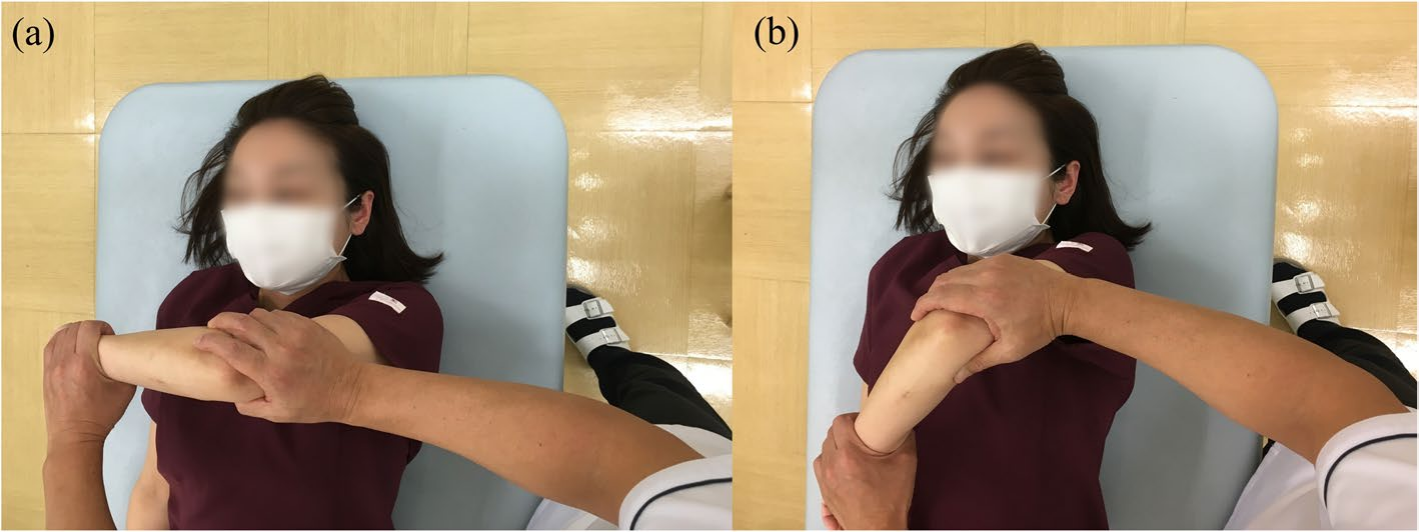
Horizontal adduction (**a**) to internal rotation (**b**) of the shoulder joint

**Fig. 5:**
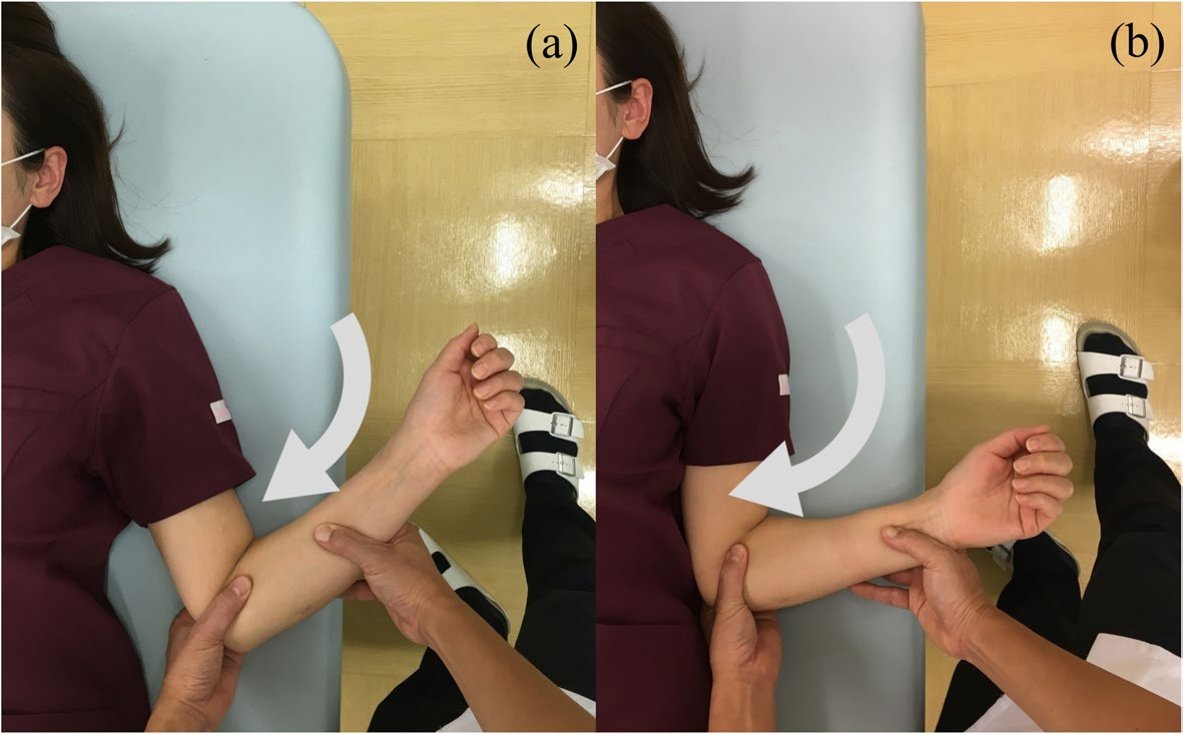
Abduction at 90°of external rotation (**a**) to adduction (**b**). Full adduction of the glenohumeral joint while preventing compensatory motions of the scapula

**Fig. 6:**
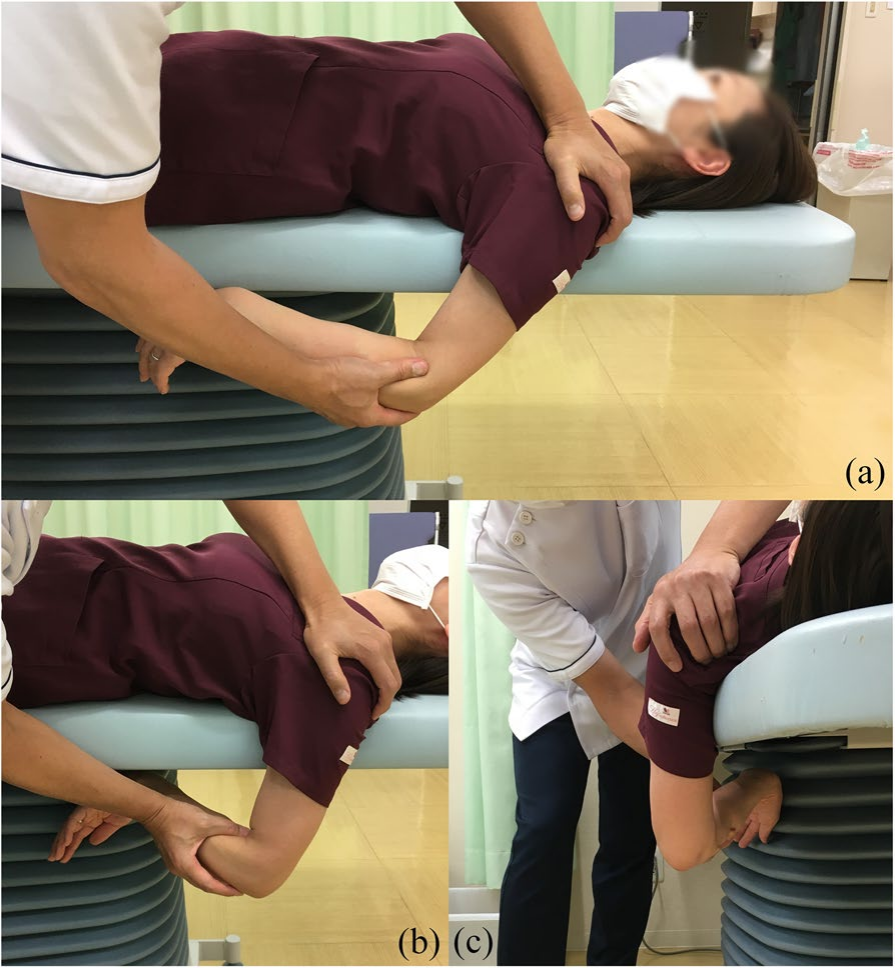
Extended position to internal rotation. The doctor presses the patient’s scapula against the bed and holds it in place while extending and internally rotating the shoulder joint

The patient is shifted to the supine position. The doctor then abducts the shoulder joint to 90° (Fig. 3a), externally rotates it by 90° (Fig. 3b), and then maximally abducts the arm (Fig. 3c). Next, the shoulder is flexed horizontally (Fig. 4a) and internally rotated from that position (Fig. 4b). The doctor then adducts the shoulder joint from the 90° of abduction and external rotation position (Fig. 5). Finally, the shoulder joint is extended (Fig. 6a) and internally rotated (Fig. 6b, c).

The entire procedure for joint manipulation is then repeated to obtain the full ROM in all directions. The patient is then required to rest for 15 min. If heart rate and blood pressure are normal and the patient does not feel unwell, they are discharged home with the arm immobilized in a triangular bandage until the effects of anesthesia have worn off.

In this study, the ROM at the shoulder joint in flexion, abduction, and external rotation was measured before silent manipulation and at 1 week and 1, 2, and 3 months after the procedure. The same anesthesiologist measured ROM visually in increments of 5° with the patient seated on the edge of the bed.

The shoulder joint is immobilized with a triangular bandage for 2–5 h until the effect of anesthesia wears off. Thereafter, there are no restrictions on daily activities. However, patients are asked to take the prescribed loxoprofen if they experience pain. Heavy lifting, tennis, golf, and other sports are prohibited for 2 months. Patients come to the hospital for physical therapy once or twice a week for approximately 3 months.

### Statistical analysis

The ROM measurements at 1 week and at 1, 2, and 3 months after manipulation were compared with those obtained before the intervention. Changes in ROM values for the shoulder joint in flexion, abduction, and external rotation at the four assessment points were compared with the corresponding values at baseline using *t*-tests with Bonferroni correction. For patients who underwent manipulation of the same shoulder joint on more than one occasion, only the initial data were used. To predict ROM improvement at 3 months after manipulation, a multiple regression analysis was performed with flexion, abduction, and external rotation as dependent variables, respectively, and with sex, age, and duration of symptoms as independent variables. All statistical analyses were performed using EZR for Windows version 1.54 (https://www.jichi.ac.jp/saitama-sct/SaitamaHP.files/statmedEN.html) [12]. Significance was set at α = 0.0167 (0.05/3) for the multiple regression analysis and at α = 0.0125 (0.05/4) for the other cases.

## Results

The study included 1,665 shoulders of 1,610 patients (519 men, 1,146 women; mean age 55.4 ± 8.8 years). The mean duration of symptoms was 6.6 ± 7.1 months (range, 1–120; no data were available for 2 shoulders). Twenty shoulders of 19 patients (6 men, 13 women; mean age 54.6 ± 8.5 years) underwent manipulation of the same shoulder joint twice. None of the patients underwent more than three manipulation procedures. In total, 1,627 shoulders were followed up at 1 week, 1,494 at 1 month, 1,169 at 2 months, and 858 at 3 months after manipulation.

**Fig. 7:**
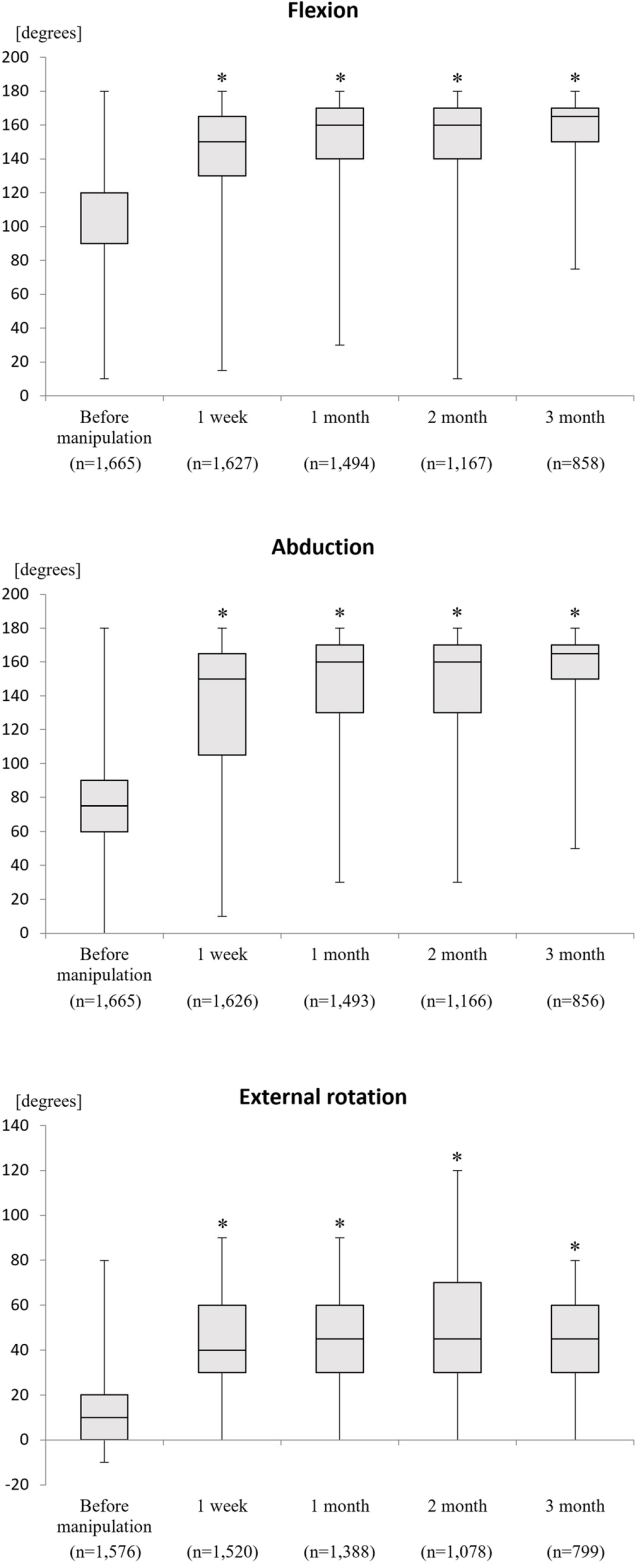
Range of motion in flexion, abduction and external rotation before and after manipulation. "n" indicates the number of shoulders evaluated. *Significant difference after silent manipulation (*p* = 0.0000)

ROM at the shoulder joint was 98.8° (95% confidence interval [CI] 97.9–99.8) before silent manipulation and 155.5° (95% CI 154.1–156.8) at 3 months after manipulation in flexion (*p* = 0.0000), 75.6° (95% CI 74.5–76.8) and 152.9° (95% 151.0–154.9), respectively, in abduction (*p* = 0.0000), and 12.7° (95% CI 12.0–13.4) and 45.9° (95% CI 44.4–47.4) in external rotation (*p* = 0.0000). ROM values were increased significantly in all three positions at all assessment times in comparison with those before silent manipulation (Fig. 7). Multiple regression analysis showed that the R-squared value of the model with flexion as the dependent variable was 0.011, with abduction was 0.001, and with external rotation was − 0.001, respectively (Table 1). There were occasional cases of transient mild respiratory distress due to phrenic nerve palsy that resolved in a sitting position. There were no complications requiring additional treatment or serious adverse events that needed urgent attention.

**Table 1:** Results of multiple regression analysis for ROM improvement

Dependent variable	Adjusted R^2^	Independent variable	Estimate	*p*-value
Flexion	0.011	Sex	5.014	0.006*
(*n* = 858)		Age	− 0.025	0.79
		Duration of symptoms	− 0.343	0.03
Abduction	0.001	Sex	2.392	0.35
(*n* = 855)		Age	0.046	0.72
		Duration of symptoms	− 0.380	0.09
External rotation	− 0.001	Sex	2.157	0.21
(*n* = 790)		Age	− 0.058	0.53
		Duration of symptoms	0.077	0.61

^*^Significant difference

## Discussion

This study retrospectively investigated changes in ROM and the risk of serious complications after silent manipulation in a large group of patients with frozen shoulder. Silent manipulation achieved early improvement of shoulder joint ROM, and the improvement was sustained over 3 months of follow-up without complications requiring additional treatment. However, this study was unable to create a model to predict ROM improvement according to sex, age, and duration of symptoms.

The detailed pathophysiology of frozen shoulder is not known. It is thought that the pathophysiologic process starts in the coracohumeral ligament, which is the roof of the rotator interval [13, 14]. The initial symptom is limited external rotation of the shoulder due to contracture of this ligament. In the advanced stages, the glenohumeral joint capsule becomes thickened and contracted, further limiting ROM in all directions [13, 14].

We have found that silent manipulation of the shoulder joint significantly improves ROM. We perform maximal abduction and adduction of the shoulder in the external rotation position. We believe that the adhesions between the coracohumeral ligament and the rotator interval are released by the forced external rotation, resulting in improved ROM in external rotation followed by improved ROM in abduction and flexion. Silent manipulation aims to both increase ROM and improve function at the shoulder joint. Movements that are impaired by frozen shoulder include those performed with the hands behind the back, such as putting on a brassiere or threading a belt. Therefore, we also perform manipulation to improve the ROM of internal rotation when the shoulder is in an extension position.

A study that summarized the results of meta-analyses of randomized controlled trials comparing conservative treatments for frozen shoulder reported that hydrodilatation with corticosteroids was the most effective treatment [15]. Hydrodilatation improves the contracture and pain associated with adhesive capsulitis by injecting a sufficient amount of fluid in the shoulder capsule [16]. However, it is unlikely that injection of fluid will eliminate all adhesions and improve the entire ROM at the shoulder joint. In a previous study [17], hydrodilatation improved the flexion angle by approximately 20° at 6 weeks after the procedure and by approximately 20°–30° at 12 weeks. However, in the present study, the flexion angle was improved by about 45° at 1 week and 65° at 3 months after treatment. Therefore, we believe that silent manipulation is a better treatment for frozen shoulder.

This report is the first to summarize the effects of manipulation with conduction anesthesia for frozen shoulder in such a large group of patients. Conservative therapy requires long-term rehabilitation, which places a heavy psychological burden on the patient [18]. Manipulation under general anesthesia incurs heavy physical and financial costs for the patient [19]. In Japan, general anesthesia costs about $460 (60,000 yen), while conduction anesthesia costs only about $13 (1,700 yen). A randomized trial of manipulation under general anesthesia, arthroscopic capsular release, and physiotherapy found that manipulation under anesthesia was the most cost-effective, although none of the three interventions were clinically superior [20]. We have used silent manipulation in the hope of removing these burdens. Several previous studies have reported on the safety of conduction anesthesia and the effectiveness of manipulation [8, 9, 11]. A magnetic resonance imaging study in 30 patients who underwent manipulation under an ultrasound-guided cervical nerve root block found a significant improvement in pain with no fractures [21]. However, one case was reported in which an avulsion fracture of the inferior glenoid rim occurred during the manipulation [10]. Therefore, we believe that silent manipulation is a safe and effective treatment, but it must be performed carefully by an experienced physician because of the risk of serious albeit rare complications.

This study has several limitations. First, it was performed retrospectively using data from medical records, which meant that pain, function, history, and complications could not be adequately assessed. Second, the long-term clinical outcomes for the patients are unknown because consultations held 3 months after manipulation were voluntary only. We assume that the 23 patients who stopped coming to the clinic had improved. Third, almost all shoulders had obtained 180° ROM in flexion and abduction under anesthesia but were immobile in full flexion and abduction postoperatively, and shoulder contracture recurred in 1.2% of shoulders (20/1665), requiring a second manipulation procedure. Therefore, we believe that rehabilitation is necessary after silent manipulation.

## Conclusions

Silent manipulation resulted in rapid improvement in ROM at the shoulder joint after the procedure, and the effect was maintained 3 months later. Therefore, silent manipulation should be an effective treatment for patients with shoulder contractures.

## Supplementary Information

**Additional file 1.**

**Additional file 2.**

## Data Availability

The datasets analyzed in this study are available from the corresponding author on reasonable request.

## Abbreviation

ROM: Range of motion
